# 

**Published:** 1911-01

**Authors:** 


					﻿Vol. XXVI.
JANUARY, 1911.
No. 7.
TE X
MEDICAL
JOURNAL
“Red Back”
PUBLISHED AT AUSTIN, TEXAS, BY
F. E. DANIEL, M. D., - - - Editor and
PRINTED BY THE VON BOECKMANN-JONE8 «O.
Subscription, 81.00 a Year, in Advance. -	-	-	- Single Cinder WCents
Entered at the Postoffice at Austin, Texas, as second class mail matter.
				

